# Midwifery Leadership in a Changing World—Why Is This So Challenging? A Reflective Commentary

**DOI:** 10.3390/healthcare13192473

**Published:** 2025-09-29

**Authors:** Marie Lewis

**Affiliations:** Lewis M Consultancy Ltd., Powys SY21 8EA, Wales, UK; lewismarie@live.co.uk

**Keywords:** midwifery leadership, maternity services, compassionate leadership, courageous leadership, governance, workforce, Safety-II, psychological safety

## Abstract

**Background:** Midwifery leadership is central to delivering safe, high-quality maternity care. Yet despite sustained investment in leadership development and governance frameworks, UK national reviews consistently identify leadership as a weakness. Understanding why this persists is vital to achieving meaningful improvement. **Objective:** This paper offers a reflective commentary on the challenges of midwifery leadership in the UK, drawing on national evidence, leadership theory, and professional experience. **Methods:** A reflective commentary approach was adopted, informed by over 30 years of practice across clinical, academic, and national improvement roles. The discussion integrates insights from national maternity inquiries, academic literature, international comparisons, and leadership theories including compassionate, courageous, and adaptive leadership. **Findings:** Structural and cultural barriers—including workforce shortages, rising clinical complexity, tensions between midwifery- and medically led models of care, and punitive governance systems—limit the effectiveness of midwifery leadership. These conditions erode psychological safety, fuel attrition, and constrain succession planning. Reflection on professional experience highlights the impact of these dynamics on leaders’ ability to act with confidence and influence. Evidence also points to the value of relational, values-based behaviours—compassion, courage, adaptability, and systems thinking—in enhancing resilience and outcomes. International examples show that supportive policy environments and greater autonomy enable midwifery leadership to thrive. **Conclusions:** Midwifery leadership requires both individual capability and structural support. Practical priorities include dismantling punitive cultures, embedding Safety-II approaches, investing in leadership development, and enabling professional autonomy. Without such systemic reform, the ambitions of the NHS Long Term Plan will remain at risk, regardless of individual leaders’ skills.

## 1. Introduction

Midwifery leadership is widely recognised as central to the delivery of safe, high-quality, and compassionate maternity care. Effective leadership empowers staff to reach their full potential, promotes professional autonomy, and fosters cultures of psychological safety and person-centred values. When these conditions are in place, midwives are more likely to feel supported, engaged, and able to provide the best possible care to women, birthing people, and families.

Yet, despite sustained investment in leadership development, the UK continues to face significant challenges in embedding strong and effective leadership within maternity services. Several national inquiries, including Ockendon (2022) [1] and Kirkup (2022) [2], have exposed leadership failings as contributory factors to avoidable harm. Comparable concerns have been raised in Scotland, Wales, and Northern Ireland, where reviews have identified weaknesses in governance, assurance, and leadership oversight. These findings point to a systemic and long-standing problem rather than isolated organisational issues. In the context of increasing clinical complexity, workforce shortages, and growing public scrutiny, understanding what limits effective leadership have become a public health priority.

Although there has been considerable investment in leadership training through national programmes such as the NHS Leadership Academy and the Florence Nightingale Foundation, as well as local clinical leadership initiatives, persistent gaps remain. Uncertainty continues about what “good midwifery leadership” looks like in practice, how leaders can balance the dual demands of leadership and management, and how cultural and structural conditions shape their ability to act. Leadership and management, though conceptually distinct, are interdependent in practice: leadership is associated with vision, influence, and inspiring others, while management is concerned with planning, organising, and delivering services effectively. Both are essential in maternity settings, where staff must navigate strategic priorities, operational pressures, and the emotional realities of frontline care. However, within maternity cultures, “management” is sometimes stigmatised, and senior staff may distance themselves from this function, stating “I am not a manager, I am a leader.” This false dichotomy can weaken organisational capacity by overlooking the need for agility—the ability to move fluidly between leadership and management roles depending on the context.

In parallel, the academic and professional development pathway for midwives has evolved significantly. The transition from diploma to degree-level registration, alongside greater uptake of postgraduate education, has raised the educational profile of the workforce. Specialist MSc programmes in advanced clinical practice and midwifery leadership are increasingly available, supporting the growth of expert clinical leaders. At the same time, rising clinical and social complexity has driven the creation of specialist midwifery roles, for example, in bereavement care, safeguarding, substance misuse, perinatal mental health, and public health. These roles bring additional leadership responsibilities and embed midwives more deeply within governance and improvement structures. Emerging evidence suggests that higher education levels are linked to increased leadership confidence and more effective clinical decision-making [3,4]. Yet despite these advances, national reviews continue to report poor leadership practices, raising enduring questions about whether leaders are “born” or “made,” and how best to cultivate the skills required for effective leadership in maternity care.

Moreover, governance and assurance frameworks such as the Maternity Incentive Scheme (England), the Maternity and Neonatal Safety Support Programme (England and Wales), and the Perinatal Quality Network (Scotland) have been introduced to strengthen safety. However, these initiatives risk contributing to bureaucratic, punitive cultures that undermine leadership. Research suggests such environments can create fear, moral injury, and disempowerment among senior midwives, contributing to attrition and weakening succession planning [2,5].

This reflective commentary explores why leadership in UK maternity services remains so challenging. Drawing on national evidence, leadership theory, and over three decades of professional practice across clinical, academic, and national improvement roles, the paper considers the cultural, structural, and personal dimensions that shape leadership in maternity care. Its aim is to identify the barriers that constrain midwifery leadership and highlight practical strategies for improvement. It asks: what does it take to be an effective midwifery leader in these complex and challenging times?

## 2. Methodology

This study adopts a reflective commentary design to examine the characteristics, challenges, and contextual influences shaping midwifery leadership in the United Kingdom. Reflective Praxis provides an established approach for integrating empirical and policy evidence with practice-based insight to illuminate complex, underexplored issues and their real-world implications [6]. The method enabled critical engagement with the literature alongside contextual interpretation grounded in professional experience.

### 2.1. Identification of Sources (Databases, Organisations, Timeframe)

Evidence was identified through purposive searches of CINAHL and MEDLINE, supplemented by targeted searches of key organisational websites (NHS England, Royal College of Midwives, Royal College of Obstetricians and Gynaecologists, Care Quality Commission, National Perinatal Epidemiology Unit/MBRRACE-UK, Healthcare Improvement Scotland, Regulation and Quality Improvement Authority, Welsh Government, NICE, The King’s Fund), and reference list hand-searching.

Search terms included midwifery leadership, compassionate leadership, courageous leadership, adaptive leadership, safety culture, Safety-II, maternity care, professional autonomy, workplace culture, succession planning, governance.

Timeframe: 1990–August 2025 to capture historical developments and contemporary policy/culture.

Geographic focus: UK sources were prioritised; selected international comparators were included where they offered relevant contrasts on autonomy, governance, or leadership frameworks.

### 2.2. Screening, Eligibility and Inclusion/Exclusion

Inclusion criteria:

(i) Peer-reviewed research (empirical or theoretical) on leadership/culture/governance in maternity or closely related healthcare contexts.
(ii) National inquiries, official policy/guidance and professional reports directly relevant to maternity leadership, governance, or culture.
(iii) Authoritative texts/monographs shaping leadership discourse in healthcare.

Exclusion criteria: opinion pieces lacking citations, non-healthcare contexts, non-English publications, and duplicate records.

Because this is a reflective commentary (not a systematic review), screening was pragmatic, and relevance-driven. Titles/abstracts were reviewed for fit; potentially relevant full texts were checked against the inclusion criteria.

Included set (non-exhaustive but sufficient for argumentation): n = 50 sources comprising

- Peer-reviewed journal articles: n = 11.
- National inquiries/policy/professional guidance & reports: n = 33.
- Books/monographs or book chapters: n = 6.

(See Table 1 for a summary by source type with examples drawn from the reference list.)

### 2.3. Integration of Reflection and Steps to Manage Subjectivity

Reflective analysis drew on the author’s 30 years of UK maternity leadership across clinical, academic, and national improvement roles. Reflection was integrated through an iterative process to

1. Interpret evidence in context (e.g., how policies played out operationally);
2. Surface tensions (leadership–management balance; autonomy vs. assurance);
3. Generate practice-oriented insights and feasible implementation steps.

To limit subjectivity, three safeguards were applied:

- **Traceability:** reflective statements are explicitly linked to cited evidence in-text (signposted as “reflection” where appropriate), ensuring insights illustrate rather than replace empirical/policy findings.
- **Triangulation of source types:** journal evidence was considered alongside national inquiries and policy frameworks to cross-check interpretations.
- **Deliberate separation in reporting:** sections distinguish evidence-derived themes from reflective insights (e.g., in Findings/Discussion and Conclusion).

### 2.4. Transparency Aids (Summary Table)

A summary table (Table 1) is provided to illustrate the spread of included sources (n = 50). Because this is not a systematic review, intermediate attrition counts (e.g., at title/abstract vs. full-text stages) were not recorded prospectively; the table is presented to illustrate the process, not to imply PRISMA-level auditing. Additional texts known to the author through her day-to-day work have also been cited.

## 3. The Changing Context of Maternity Services

The history of midwifery is marked by ongoing struggles for professional autonomy and recognition, and these tensions continue to shape midwifery leadership pathways today. In the UK, while midwifery is protected by statute as an autonomous profession, the legacy of medical dominance in maternity care has led to persistent power imbalances between midwives and obstetricians. The 20th-century institutionalisation of birth shifted maternity care from homes to hospitals, embedding a medically led, risk-averse model that often marginalised midwifery-led approaches [30,32]. Although significant efforts have been made to reassert midwifery autonomy—most notably through the Changing Childbirth (1993) report [19] and more recently Better Births (2016) [23]—midwives continue to face barriers to practising fully autonomously within hierarchical NHS structures [10,33]. Amidst the context of social media campaigns and media reporting of harm caused by maternity services and a widening discourse between midwifery led physiological and medical led high tech ideologies.

These power dynamics not only influence clinical practice but also impact leadership development [12]. Midwifery leaders are often required to act as boundary-spanners, advocating for midwifery philosophy and evidence-based, person-centred care within systems that prioritise performance metrics and biomedical risk management [14,17]. The tension between professional values and system expectations can make leadership roles politically and emotionally demanding, contributing to burnout and attrition [7]. Furthermore, pathways into leadership are shaped by structural limitations—such as lack of access to strategic decision-making forums and limited investment in midwifery-specific leadership development.

International comparisons further illuminate this challenge. In the USA, midwives face significant restrictions on scope of practice in many states due to regulatory and institutional barriers, resulting in limited autonomy and integration within the healthcare system [9]. In contrast, countries such as New Zealand and the Netherlands offer models of care where midwives enjoy high levels of autonomy and professional status, supported by strong governance structures and a policy environment that values physiological birth and continuity of carer [8,13,31,37,38]. These models demonstrate how greater midwifery autonomy and structural support can enable leadership to flourish, influence service design, and improve outcomes [29,39].

In the UK, midwifery leadership must therefore be understood not only as a function of individual capability but also as a political act: the assertion of professional values within a system that continues to challenge the authority and independence of midwifery [40,41]. Recognising this context is essential if we are to develop leadership pathways that truly support midwives to lead with authenticity, courage, and influence [42].

The context in which maternity services are provided is evolving rapidly, shaped by demographic, social, and systemic challenges. There is increasing complexity within the population, with rising levels of obesity, diabetes, mental health conditions, and socio-economic deprivation impacting pregnancy outcomes [20,43]. These trends underscore the growing need for multi-disciplinary, cross-boundary collaboration across health, social care, and community sectors to ensure safe and effective care.

Despite these challenges, public and professional expectations remain high. Families increasingly anticipate a risk-free birth experience with positive physical and emotional outcomes [18]. However, population health indicators, such as maternal obesity and smoking in pregnancy, continue to increase in some areas, and interventions aimed at addressing these trends have achieved only limited success [44,45,46].

At the same time, there are significant barriers to providing the very elements of care that are known to improve holistic outcomes. Models such as continuity of career, which evidence shows reduces preterm birth, improves satisfaction, and enhances safety, have been scaled back or discontinued in many areas due to staffing shortages [15]. Similarly, closures of alongside and freestanding birth centres and a reduction in midwifery-led care have reduced choice for families [27]. Postnatal care and breastfeeding support remain inconsistent, often compromised by workforce pressures and resource constraints [25]. The time for midwives to listen and provide relational care is increasingly replaced with technology-driven processes and bureaucratic documentation requirements [47].

In stark contrast, clinical intervention rates continue to rise. Caesarean section rates in England reached almost 35% in 2022, and induction of labour rates now exceed 40% in many regions [20]. While these interventions can be lifesaving, they are associated with higher short-term risks and long-term implications for maternal health and future pregnancies, which are not yet fully understood. This shift demands more technology-dependent services, increased costs, and highly skilled staff—creating additional strain on already stretched resources [48,49].

The complexity of cases has driven a need for more specialist midwifery roles, such as safeguarding, perinatal mental health, and continuity leads, often consolidated into fewer sites to maintain expertise and safety [26]. Financial pressures exacerbate these challenges, compelling leaders to make difficult decisions about service configuration, including shared commissioning arrangements and group service models [21]. This creates a widening tension between promoting midwifery-led, community-based care and responding to the escalating demands of high-risk, hospital-based care.

## 4. Reflective Component: Linking Leadership Theory to My Experience

Reflecting on my career, I recognise that many of the leadership qualities identified in current evidence—such as compassionate, courageous, and adaptive leadership—have been central to my practice, often under challenging circumstances. With over 30 years in the NHS, my roles have spanned from integrated midwife to Consultant Midwife, Associate Director of Research, and most recently, National Maternity Improvement Advisor. These experiences have shaped my understanding that leadership in maternity is not only about strategic planning and governance but also about human connection, moral courage, and adaptability in the face of complexity.

Compassionate leadership, as described by West [36] and supported by evidence in Compassionomics [50], resonates deeply with my personal philosophy of care. In my role as Consultant Midwife, I led quality improvement initiatives—such as setting up a rural midwife-led day assessment service and introducing digital maternity records—by engaging staff through listening, empathy, and shared purpose. I have always prioritised creating environments where midwives feel valued and safe to share ideas, recognising that staff wellbeing directly impacts the experience of women and families.

However, my leadership journey has also demanded courage. During my tenure as National Maternity Improvement Advisor, I worked with trusts rated as “requires improvement” or “inadequate” by the CQC, where cultures of fear and silence were prevalent. Leading improvement in these contexts required challenging entrenched behaviours, addressing resistance, and advocating for transparency even when these conversations were uncomfortable. This aligns with the principles of courageous leadership [51,52], which stress the importance of moral courage in protecting patient safety and driving change [53,54].

Adaptability has been another critical competency in my career. The COVID-19 pandemic exemplified this, when I led a Trust-wide staffing response hub, balancing operational pressures with staff redeployment and wellbeing. Similarly, working internationally with the Florence Nightingale Foundation to review midwifery models of care in New Zealand and Denmark strengthened my ability to think systemically and integrate global best practices into local improvement plans.

These experiences reinforce my belief that effective midwifery leadership is about balancing compassion with courage, and strategic vision with relational care. In a climate of increasing complexity, media scrutiny, and regulatory pressure, my commitment remains to lead in a way that empowers teams, upholds professional values, and keeps women and birthing people at the centre of every decision.

## 5. The Changing Workforce in Maternity and Leadership Challenges

The maternity workforce is undergoing significant demographic and structural change, presenting unique challenges for leadership. The midwifery profession in the UK is ageing, with almost 50% of midwives aged over 45, creating concerns about retirement-related attrition and loss of expertise [22]. At the same time, there has been an influx of newly qualified midwives and international recruits to address persistent workforce shortages [55]. This creates a generational gap within teams, often with contrasting expectations, values, and working styles. Younger midwives frequently prioritise work–life balance, flexibility, and career progression, while more experienced staff may emphasise traditional hierarchies and continuity of care [28,56,57,58]. Leaders must navigate these differing perspectives while maintaining team cohesion and service quality.

Moreover, the increasing reliance on internationally educated midwives introduces cultural diversity and the need for inclusive leadership practices, particularly in supporting adaptation to UK clinical standards and professional culture [22]. These dynamics occur against a backdrop of chronic workforce shortages—England alone has an estimated shortfall of over 2500 full-time equivalent midwives [55]. The pressure to fill gaps often results in redeployment, loss of continuity models, and reduced professional development opportunities, further challenging morale, and retention [24]. For leaders, balancing service delivery with staff wellbeing, professional development, and equitable treatment across diverse workforce groups is an increasingly complex task that requires adaptive, compassionate, and culturally competent leadership [36].

Midwifery leaders find themselves navigating these competing priorities while managing day-to-day operational pressures, workforce shortages, and the need to uphold women’s and birthing people’s values and choices. Simultaneously, they face intensifying scrutiny from regulatory bodies, escalating assurance and data requirements, and a media landscape often critical of maternity services and the midwifery profession [1,2]. These dynamics raise a fundamental question: **what does it take to be an effective midwifery leader in these challenging and complex times?**

## 6. A Culture of Fear in Maternity Leadership

The culture within maternity services has shifted significantly in recent years, and for many in the workforce, fear has become a dominant influence on how they lead and practice. Fear of getting things wrong, fear of blame, fear of referral to the Nursing and Midwifery Council (NMC), and fear of losing employment now shaped day-to-day decision-making for midwifery leaders [1,2]. This fear is compounded by the increasing prevalence of media scrutiny and public naming and shaming when adverse events occur [18]. Leaders often describe the emotional toll of carrying responsibility for both patient safety and organisational reputation while trying to protect their teams [11].

Underlying this is the very real fear of causing harm to women, birthing people, and babies—the fundamental drivers for why midwives enter the profession. However, this moral responsibility, when combined with punitive regulatory systems and the constant demand for compliance with assurance processes, can create an overwhelming sense of vulnerability among senior staff [7]. Reports such as the East Kent Inquiry [2] have highlighted how a blame-focused culture can become embedded, leading to defensive practice and diminished psychological safety within teams.

Being a senior leader in maternity today can feel like a no-win situation, with high expectations and limited resources creating the perfect conditions for stress and burnout. Research indicates that midwifery leaders experience higher rates of stress compared to many other health professionals, with workload, fear of litigation, and responsibility for serious incidents frequently cited as primary stressors [16]. These pressures contribute to experienced leaders leaving the profession after short tenures in senior roles, resulting in a loss of expertise at a time when it is most needed [55]. In turn, younger and less experienced midwives are often placed in leadership roles with limited preparation, mentorship, or organisational support, leaving them vulnerable to the same cycle of stress and attrition [55].

The consequence is a system where fear becomes normalised and “leading through fear” starts to feel inevitable. This environment undermines the principles of compassionate and transformational leadership advocated in NHS policy [59] and erodes the psychological safety that is essential for learning and improvement [36]. If this trajectory continues, the profession risks perpetuating a culture that prioritises compliance over curiosity and fears over innovation—conditions that are incompatible with sustainable improvement in maternity care.

## 7. What Makes a Good Midwifery Leader in These Challenging Times?

Compassionate leadership remains central to creating positive cultures in healthcare. West [36] emphasize that compassionate leadership—defined by attentiveness, understanding, empathic response, and helpful actions essential for improving staff wellbeing and patient outcomes. This is strongly supported by emerging evidence from *Compassionomics*, which demonstrates that compassion in healthcare is not only a moral imperative but also improves clinical outcomes, reduces costs, and enhances staff engagement [50]. In maternity services, where relational care and emotional labour underpin practice, compassionate leadership provides a crucial buffer against stress, moral injury, and the fear-based behaviours associated with punitive or compliance-driven cultures. Leaders who listen deeply to staff concerns, respond with empathy, and take meaningful action foster trust, psychological safety, and resilience within their teams—conditions that translate into safer, more respectful care for women and birthing people.

However, compassion alone is not enough. Good midwifery leaders must also demonstrate courageous leadership, which involves speaking truth to power, challenging behaviours, and dismantling systems that perpetuate harm, bias, or inequity [60,61]. Courageous leadership requires moral courage—the ability to act ethically and advocate for what is right, even in the face of personal or professional risk. Evidence suggests that leaders who demonstrate courage foster greater trust, transparency, and team resilience, particularly in high-stakes environments such as maternity services [54]. Recent inquiries, including Ockenden (2022) [1] and Kirkup (2022) [2], highlighted the devastating consequences of cultures where staff feel silenced and unable to escalate concerns. Both reports recommend the creation of psychologically safe environments, where leaders actively encourage speaking up and ensure staff are protected from blame or retaliation. Such leadership behaviours are essential for preventing failures and promoting a culture of learning and accountability.

Brené Brown [34] argues that true leadership requires vulnerability, courage, and the willingness to step into uncertainty despite the risk of criticism or failure. For maternity leaders, this is particularly critical in the current climate, where political, financial, and media pressures often drive priorities that may conflict with what is right for women and birthing people. Brown emphasises that “courage is contagious” and that leaders who model brave decision-making create cultures of integrity and trust. In maternity care, this means advocating for person-centred models, such as continuity of carer and midwifery-led services, even when these approaches face resource constraints or policy challenges. Courageous leadership in this context is about resisting the temptation to comply passively with top-down demands and instead holding firm to evidence-based principles that improve safety, choice, and experience. Such bravery is not about avoiding fear but about “choosing courage over comfort” [34], a stance that is essential if maternity services are to fulfil their purpose of safeguarding the wellbeing of women, birthing people, and families.

Adaptability and systems thinking are also critical. The complexity of modern maternity care—with rising intervention rates, workforce shortages, and increasing specialisation—demands leaders who can navigate ambiguity, respond to rapid change, and integrate perspectives across professional and organisational boundaries. This includes using data intelligently to inform decisions, while avoiding the trap of prioritising metrics over meaning. In this context, the shift towards a *Safety-II* approach offers a valuable framework for rethinking safety and improvement. Unlike traditional *Safety-I* thinking, which focuses on what goes wrong and preventing errors, *Safety-II* encourages leaders to understand and reinforce what goes right in everyday practice by examining how healthcare professionals adapt successfully to variability and complexity [62]. Applying *Safety-II* principles in maternity settings promotes a more holistic understanding of quality and resilience—focusing not only on avoiding harm but also on fostering the conditions under which safe and effective care routinely occurs. This systems-oriented perspective aligns closely with adaptive leadership, empowering midwifery leaders to move beyond compliance-driven models and instead cultivate learning cultures that value professional judgement, reflection, and innovation.

Tom Peters, a leading voice in leadership theory, advocates for the principle of “humanizing leadership,” emphasizing that effective leaders must recognise the humanity of those they serve and those they lead [35]. His argument centres on the belief that leadership is fundamentally about relationships, empathy, and valuing human behaviour over systems or metrics. For maternity leaders, this philosophy is critical. The current climate, shaped by political pressure, media scrutiny, and financial constraints, often prioritises compliance and cost-efficiency over person-centred care. Yet maternity care is, by its very nature, relational and deeply personal. Leaders who adopt a humanising approach focus on what truly matters for women and birthing people—their safety, dignity, and experience—rather than simply responding to external demands. By creating compassionate, psychologically safe environments for staff and resisting a culture driven solely by performance targets, maternity leaders can align care with core professional values and uphold the principles of respectful, individualised maternity care. Embracing Peters’ vision means re-centring leadership on humanity, enabling leaders to balance organisational pressures with the moral imperative to do what is right for families.

Finally, effective midwifery leaders must be committed to developing others. The NHS People Plan and Maternity Transformation Programme emphasise the importance of talent development and succession planning. Investing in structured leadership development programmes ensures that future leaders are equipped to handle the demands of contemporary maternity care.

## 8. Limitations

As a reflective commentary, this paper does not aim to provide a comprehensive or systematic synthesis of all available evidence on midwifery leadership. The selection of literature was purposive and interpretive, guided by relevance to the UK context and alignment with the author’s professional experiences. This may introduce selection bias and limit generalisability. The integration of reflective analysis adds depth and authenticity but also introduces subjectivity, as insights are shaped by the author’s individual leadership journey. While the inclusion of policy documents strengthens the practical relevance of the review, it may lack the methodological rigour associated with formal empirical research. This is not a systematic review, and findings should be interpreted cautiously. Future research using systematic methods or empirical inquiry could build on this work to further test and expand the themes identified.

## 9. Results/Discussion

In a climate of rising complexity, scrutiny, and workforce pressure, effective midwifery leadership is a public health priority. Evidence from national reviews demonstrates that command-and-control approaches are inadequate; leadership failures contribute to avoidable harm. The following sections explicitly frame barriers, enablers, and a roadmap, supported by an international comparison and implications for research.

### 9.1. Barriers (Summary Table 2)

Below is a table outlining the main barriers to achieving good midwifery leadership and how that is identified in practice.

### 9.2. Enablers (Summary Table 3)

Below is a table outlining the main enablers to achieving good midwifery leadership and how that is achieved in practice.

### 9.3. Roadmap for Implementation

Phase 1: 0–3 months (Foundations)

1. Name the work: Publish a one-page case for change (barriers/enablers), with 3–5 success measures.
2. Protect time: Agree minimum 0.1–0.2 WTE leadership time per Band 6–8a; lock into rosters.
3. Governance reset: Add a Safety-II “what went right” item and a 10-min “learning huddle” to every governance meeting.
4. RACI the essentials: Map ownership for incidents, guidelines, audits, and escalation; publish owners and Specified Learning Actions.

Phase 2: 3–12 months (Build & integrate)

5. Tiered development: Deliver a Band 6–8a pathway (foundations → leading teams → leading services) aligned to NHS Leadership Model behaviors.
6. Mentor & deputies: Assign mentors; formalize deputy roles with objectives; track progression quarterly.
7. Community of practice: Set up a monthly midwifery–obstetric–neonatal improvement forum with a shared run-chart pack.
8. Reduce bureaucracy: Retire or merge low-value meetings; aim for a 20% reduction in duplication/wasted time.

Phase 3: 12–24 months (Embed & scale)

9. Autonomy contracts: Agree local decision rights for midwifery-led pathways; monitor utilization and safety.
10. Outcomes focus: Tie leadership behaviors to appraisal; link to quality and safety climate, retention, and experience measures; publish a simple annual leadership impact report.

Minimum evaluation set (quarterly): using appreciative enquiry to learn from what is going well, as well as areas for improvement, including quality and safety climate (short scale), staff retention/turnover, sickness absence, incident learning completion, % actions closed on time, patient-experience and staff experience “communication & involvement” items.

### 9.4. International Comparison: What Is Transferable? (Table 4)

Below is a summary table showing the transferable features noted from the authors international comparison and the likely barriers to applying the learning in the UK.

**Bottom line:** The most transferable elements are leadership behaviors, Safety-II learning practices, clear decision rights for midwifery-led care, and cross-boundary communities of practice. The main blockers are workforce capacity, medico-legal/assurance expectations, and digital interoperability, addressable through phased pilots, agreed decision contracts, and removal of low-value bureaucracy.

### 9.5. Implications for Future Research (Specific Questions)

1. Safety-II & wellbeing: How does introducing Safety-II learning huddles affect staff psychological safety, moral injury markers, and incident-learning quality in maternity teams over 12 months?
2. Protected leadership time: What is the impact of 0.1–0.2 WTE protected leadership time for Band 6–8a on action closure rates, escalation timeliness, and staff retention?
3. Leadership–management integration: Does implementing RACI plus monthly PDSA cadences improve execution (on-time delivery, duplication reduced) and perceived role clarity?
4. Communities of practice: Do cross-professional CoPs reduce escalation delays and increase adherence to shared protocols?
5. Succession & mentoring: What mentoring models most effectively build a Band 6–8a leadership pipeline and reduce time-to-competence in new postholders?
6. International transferability: Which elements of NZ/NL autonomy models are feasible within NHS assurance requirements, and what adaptations sustain safety and equity?

## 10. Conclusions

By clarifying barriers and enablers, committing to a phased roadmap, and testing what works through targeted research, services can move beyond describing “good leadership” to building it in practice. This combination—behavioural standards, protected time, Safety-II learning, decision rights, and mentoring—creates the conditions in which midwifery leaders can deliver the compassionate, courageous, and adaptive leadership needed to realise the ambitions of the NHS Long Term Plan.

## Figures and Tables

**Table 1:** Summary of included sources by type (illustrative examples).

Source Type	Count (n)	Illustrative Examples *
Peer-reviewed journal articles	11	Abdhul-Rahim et al., 2024 [4], Deery & Hunter 2010 [7]; Dixon et al., 2023 [8]; Herndon and Vanderlaan 2024 [9]; Hunter 2004 [10]; Hunter & Warren 2014 [11]; Elliott-Mainwaring 2022 [12], Foster et al., 2021 [5], Farry et al., 2025 [13]; Maude et al., 2022 [3], Rost et al., 2024 [14]; Sandall et al., 2016 (Cochrane) [15]; Sheen et al., 2020 [16]; Timothy et al., 2025 [17].
National inquiries, policy, guidance & professional reports	33	Birthrights 2025 [18]; DOH changing childbirth 1993 [19], Ockenden 2022 [1]; Kirkup 2022 [2]; NMPA 2023 [20]; NHS England (People Plan 2020/21 [21]; workforce progress 2022 [22]; Better Births 2010 [23], continuity of carer 2022 [24]; RCM briefings (2022–2023) [25,26]; CQC 2023 [27]; Sills for care [28]; WHO 2024 [29]
Books/monographs/chapters	6	Donnison 1988 [30]; Grant and Thomas 2019 [6], Guilliland and Pairman 2010 [31], Tew 1998 [32]; Page & McCourt 2005 [33]; Brown 2018 [34]; Peters 2018 [35]; West 2021 [36]

* Examples correspond to items in the reference list; exact numbering will follow the journal’s Vancouver order of citation.

**Table 2:** Barriers.

Barrier	What It Looks Like in Practice	Primary Level(s) Affected	Illustrative Indicators to Monitor
**Punitive culture & low psychological safety**	Escalation avoided; retrospective blame; defensive documentation	Team, service, organisation	Safety climate/“speaking up” survey scores; incident reporting mix (near-miss vs. harm); PSIRF learning completion rates
**Workforce shortages & attrition**	Persistent vacancies; redeployments; curtailed continuity models	Service, organisation, system	Vacancy/turnover; sickness absence; use of bank/agency; training cancellations
**Leadership–management confusion**	“I’m a leader, not a manager”; poor follow-through on plans	Individual, team, service	% objectives with owners/timelines; appraisal quality; QI project completion
**Fragmented governance & bureaucracy**	Form-filling outweighs learning; duplication across forums	Service, organisation	Meeting-to-action ratios; duplication identified/removed; cycle-time from incident to action
**Limited autonomy for midwifery leadership**	Decisions escalated by default; little scope to tailor models	Service, organisation	% decisions made at appropriate level; midwife-led pathway utilisation
**Inadequate succession planning**	Acting roles without mentoring; thin Band 6–8a pipeline	Team, organisation	Coverage of mentoring; % roles with deputies; internal fill rate for posts

**Table 3:** Enablers.

Enabler	What to Do Now (Practical Actions)	Expected Early Signals (3–6 Months)
**Compassionate & courageous leadership**	Adopt behavioural standards in appraisal; leaders’ model “listen–learn–act” huddles	Improved team safety-climate items; increased speaking-up activity without retaliation
**Safety-II & systems thinking**	Add “What went right?” to governance; run monthly success debriefs	Balanced incident review portfolio: more proactive controls tested
**Protected leadership time**	Roster 0.1–0.2 WTE leadership time for Band 6–8a; ring-fence training days	Attendance at leadership development; reduced meeting cancellations
**Clear leadership–management integration**	RACI for core processes; mandate “plan–do–study–act” cadence	Higher action closure rates; fewer “stranded” actions
**Communities of practice**	Cross-professional forums (midwifery/obstetrics/neonatal) with shared dashboards	Fewer escalation delays: joint improvement charters agreed
**Succession & mentoring**	Pair each postholder with a deputy; monthly mentoring with objectives	Internal pipeline growth; smoother cover during absence

**Table 4:** International comparison: what is transferable?

Jurisdiction	Practice Features	Transferable to NHS?	Likely UK Barriers	Mitigations
**New Zealand**	High midwifery autonomy; continuity models; strong professional partnership	**Yes, partially** (autonomy in defined pathways; continuity in targeted cohorts)	Workforce gaps; litigation climate; commissioning constraints	Pilot continuity for priority groups; autonomy compacts; shared obstetric–midwifery governance
**Netherlands**	Networked community–hospital interfaces; risk-stratified pathways	**Yes** (integrated triage; shared protocols; home-to-hospital interfaces)	Data interoperability; role clarity at boundaries	Shared referral standards; integrated digital referrals; joint drills
**Denmark**	Strong learning culture; low bureaucracy; trust-based assurance	**Elements** (Safety-II learning sessions; simpler paperwork)	Assurance expectations; external reporting	Replace duplicative paperwork with learning artefacts; agree “assurance through learning” proofs
**USA (selected states)**	Variable autonomy; high medico-legal burden	**Limited**	Regulatory and indemnity frameworks; payment models	Focus on non-financial enablers: culture, Safety-II, leadership development

## Data Availability

No new data were created or analyzed in this study. Data sharing is not applicable to this article.
